# Processing word prosody—behavioral and neuroimaging evidence for heterogeneous performance in a language with variable stress

**DOI:** 10.3389/fpsyg.2014.00365

**Published:** 2014-04-29

**Authors:** Miriam Heisterueber, Elise Klein, Klaus Willmes, Stefan Heim, Frank Domahs

**Affiliations:** ^1^Section Neurological Cognition Research, Department of Neurology, Uniklinik RWTH AachenAachen, Germany; ^2^Faculty of Medicine, Brain Imaging Facility of the Interdisciplinary Centre for Clinical Research, Uniklinik RWTH AachenAachen, Germany; ^3^KMRC – Knowledge Media Research CenterTuebingen, Germany; ^4^Section Neuropsychology, Department of Neurology, Uniklinik RWTH AachenAachen, Germany; ^5^Department of Psychiatry, Psychotherapy and Psychosomatics, Uniklinik RWTH AachenAachen, Germany; ^6^Research Centre Juelich, Institute of Neuroscience and Medicine (INM-1)Juelich, Germany; ^7^Institute of Germanic Linguistics, Philipps UniversityMarburg, Germany

**Keywords:** word stress, fMRI, interindividual differences, segmental processing, stress processing

## Abstract

In the present behavioral and fMRI study, we investigated for the first time interindividual variability in word stress processing in a language with variable stress position (German) in order to identify behavioral predictors and neural correlates underlying these differences. It has been argued that speakers of languages with variable stress should perform relatively well in tasks tapping into the representation and processing of word stress, given that this is a relevant feature of their language. Nevertheless, in previous studies on word stress processing large degrees of interindividual variability have been observed but were ignored or left unexplained. Twenty-five native speakers of German performed a sequence recall task using both segmental and suprasegmental stimuli. In general, the suprasegmental condition activated a subcortico-cortico-cerebellar network including, amongst others, bilateral inferior frontal gyrus, insula, precuneus, cerebellum, the basal ganglia, pre-SMA and SMA, which has been suggested to be dedicated to the processing of temporal aspects of speech. However, substantial interindividual differences were observed. In particular, main effects of group were observed in the left middle temporal gyrus (below vs. above average performance in stress processing) and in the left precuneus (above vs. below average). Moreover, condition (segmental vs. suprasegmental) and group (above vs. below average) interacted in the right hippocampus and cerebellum. At the behavioral level, differences in word stress processing could be partly explained by individual performance in basic auditory perception including duration discrimination and by working memory performance (WM). We conclude that even in a language with variable stress, interindividual differences in behavioral performance and in the neuro-cognitive foundations of stress processing can be observed which may partly be traced back to individual basic auditory processing and WM performance.

## Introduction

In some languages (e.g., Czech, Finnish, Polish, Turkish, Persian, or French) main stress always falls on the same position within a word (fixed stress; for a typological overview see Van der Hulst, 1999). In those languages, no minimal pairs of words exist which do only differ in terms of their stress position. Accordingly, in fixed stress languages word stress is not contrastive and does not carry lexical information. In consequence, the processing and representation of word stress is not particularly relevant in the use of such languages. In this vein, it has been repeatedly reported that speakers of languages with fixed stress encounter difficulties when confronted with tasks requiring processing or representation of word prosody (Dupoux et al., 1997; Peperkamp et al., 1999, 2010; Mehler et al., 2004; Domahs et al., 2012, 2013a).

In contrast, other languages (e.g., English, Spanish, Russian, or German) have variable stress positions. Word stress may be contrastive, carrying lexical information. Thus, there may be minimal pairs, which only differ in their suprasegmental make-up, i.e., stress pattern, their segmental sequence being identical (e.g., German verbs *umfáhren* vs. *úmfahren*, to drive around vs. to knock over). Therefore, the processing and representation of word stress is particularly relevant in languages with variable stress and speakers of those languages are typically found to be highly sensitive to suprasegmental manipulations, showing relatively good performance in a variety of tasks tapping on word stress (Domahs et al., 2008; Molczanow et al., 2013; for a direct comparison between speakers of a language with fixed stress (French) and with variable stress (Spanish or German) see Dupoux et al., 2001, 2008; Schmidt-Kassow et al., 2011a).

However, comparing speakers of different languages typically ignores the possibility that there may be substantial interindividual variability in stress processing performance even within a given language. Thus, the present study addresses the questions whether there are interindividual differences in stress processing in a language with variable stress (German) and, if so, which neural correlates may underlie those differences. Before the details of the present study will be outlined, a brief summary of research on stress processing will be given by describing word stress assignment in German and discussing evidence on the neuronal basis of stress processing.

### Word stress assignment in german

Given that German is a language with variable stress, the stress pattern of individual words is largely unpredictable and has thus to be lexicalized (Eisenberg, 2006; Domahs et al., 2008). This lexical knowledge can be used to distinguish between the elements of minimal pairs and to activate the correct meaning related to each of the members of a minimal pair. Beyond complete lexicalization, there are some rules and regularities in German stress assignment which become apparent, when participants are asked to pronounce pseudowords or have to deal with stress violations:

- Only one of the final three syllables of a word can bear main stress (“three syllable window,” Vennemann, 1990). Thus, words can have ultimate stress (U, final syllable stressed), penultimate stress (PU, prefinal syllable stressed), or antepenultimate stress (APU, semi-prefinal syllable stressed).
- Stress assignment is influenced by syllabic structure, in particular by the syllable weight of the final syllables (Tappeiner et al., 2007; Domahs et al., 2008; Janssen and Domahs, 2008; Roettger et al., 2012) such that words with open final and/or closed pre-final syllables are predominantly stressed on the penultimate syllable. Complex final syllables typically lead to main stress on the final syllable. Antepenultimate stress is typically found, when the penult is open and the final syllable is closed.
- Main stress may be conceived as surface expression of metrical foot structure (which is determined by syllable weight) such that strong feet bear main stress. As prosodic feet are typically binary (i.e., containing two syllables which form a trochee, Knaus and Domahs, 2009), but heavy final syllables are parsed into non-branching feet, ultimate and antepenultimate stress can be seen as structurally similar in contrast to penultimate stress (Domahs et al., 2008, 2013b; Haake et al., 2013).
- Penultimate stress is the most frequent pattern in German. Féry (1998) found that 73% of German bisyllabic words are stressed on the penult. In this light, it has been debated whether in German the penultimate stress pattern can be regarded as the default (e.g., Eisenberg, 1991; Kaltenbacher, 1994; Wiese, 1996; Levelt et al., 1999) or not (Giegerich, 1985; Vennemann, 1991; Féry, 1998; Domahs et al., 2008; Janssen and Domahs, 2008; Roettger et al., 2012).

Phonetically, German word stress is marked by a combination of the following cues: duration, (global) intensity, fundamental frequency (pitch), vowel formants and voice quality (for a comprehensive overview see Lintfert, 2010). Haake et al. (2013) found a significant relationship between auditory perception of duration cues and the representation of word stress both in children with specific language impairment and in typically developing children acquiring German. Heim and Alter (2006, 2007) provided EEG evidence that context stress, e.g., in a sentence, can be used as additional information to identify stress patterns.

### The neural bases of word stress processing

There are currently only very few functional neuroimaging studies investigating the neural correlates of word stress processing (Aleman et al., 2005; Klein et al., 2011; Domahs et al., 2013b). In the study by Aleman et al. (2005) participants had to identify weak-initial and strong-initial words. The bilateral supplementary motor area (SMA) and the left inferior frontal gyrus (IFG), the superior temporal gyrus (STG) as well as the superior temporal sulcus (STS), and the insula were associated with the processing of word stress compared to a semantic control condition. In the study by Klein et al. (2011) participants were asked to solve an identity matching task with pseudowords. Processing of word stress minimal pairs as compared to segmental minimal pairs was associated with activation in a bilateral fronto-temporal network. Klein et al. (2011) suggested that there is a basic system for word stress processing in the left hemisphere, whereas the right hemisphere supports the left in case of increasing task difficulty. Domahs et al. (2013b) investigated the neural correlates of processing correctly vs. incorrectly stressed words. They observed activations of the left posterior angular and retrosplenial cortex when contrasting the processing of correct vs. incorrect stress. In the inverse contrast, bilateral STG were found to be involved. The analysis of severe vs. mild stress violations revealed activations of the left superior temporal and left anterior angular gyrus. Frontal activations, including Broca's area and its right homolog, were found when contrasting mild with severe stress violations.

With respect to interindividual differences in stress processing, Boecker et al. (1999) performed an ERP study using a word stress discrimination task. Based on the median split of the behavioral outcome, they defined two groups of participants: good and poor performers. The authors found a significant N400-effect for sequence-final words with a weak-strong pattern only in the group of good performers, but not in the group of poor performers, providing first evidence to the possibility of substantial interindividual differences in word stress processing in a language with variable stress (Dutch).

### The present study

While differences in word stress processing between speakers of languages with fixed vs. variable stress have been described repeatedly (Dupoux et al., 2001, 2008; Peperkamp et al., 2010; Schmidt-Kassow et al., 2011a,b), interindividual differences within one type of language—although observed—remained largely ignored or unexplained (Boecker et al., 1999; Peperkamp et al., 1999; Domahs et al., 2008, 2013b; Dupoux et al., 2010). In general, it has been argued that speakers of a language with variable stress should perform relatively well in word stress processing (Dupoux et al., 1997, 2001, 2008, 2010; Peperkamp et al., 1999; Schmidt-Kassow et al., 2011a). Although interindividual variance in word stress processing in German has not been the focus of previous research, such variability has been observed (albeit ignored) in adult participants in previous studies (Domahs et al., 2008, 2013b). In a recent study, (Haake et al., 2013) reported interindividual variability in word stress processing in both children with specific language impairment and typically developing children. This variance was at least partly predicted by individual perceptual processing of auditory cues related to word stress (e.g., duration).

The aim of the current study was to investigate interindividual performance differences in the processing of word stress. To this end, native speakers of German had to perform a variant of a sequence recall task, adapted from Dupoux et al. (2001; see also Haake et al., 2013). Studies on languages with fixed stress using this task have shown that when demands on working memory increase, performance of speakers of such languages in reproducing pseudoword minimal pairs (e.g., míkuta vs. mikutá) decreases disproportionately (Dupoux et al., 1997, 2001). We used a suprasegmental variant of this task to investigate interindividual heterogeneity in word stress processing in native speakers of German, a language with variable stress, while a segmental variant of this task served as a control condition. Note that speakers of German should be highly familiar with both suprasegmental and segmental features since both are essential in the use of this language.

In sum, the research questions of the present study were the following: (i) Are there substantial interindividual differences in word stress processing within a group of native speakers of German, a language where this feature is functional? (ii) Which neural correlates in functional magnetic resonance imaging (fMRI) are associated with word stress processing in good and poor performers? Following the results of previous neuroimaging studies on word stress processing (Aleman et al., 2005; Klein et al., 2011; Domahs et al., 2013b), we expected to find clusters of activated voxels in the left IFG, the bilateral superior temporal gyrus/sulcus and in the insula as well as bi-hemispheric activation in the SMA. (iii) Can predictors for interindividual variability be identified (e.g., working memory abilities and/or basic auditory processing)?

## Materials and methods

### Participants

Twenty-five right-handed native German-speaking healthy volunteers (nine female; mean age = 28.8 years, *SD* = 10.1 years) participated in the study after having given their written informed consent. The study was approved by the Institutional Review Board of the Medical Faculty at RWTH Aachen University (EK 182/06).

### Stimuli

Stimulus material consisted of trisyllabic pseudowords obeying German phonotactic constraints. The pseudowords were built from five different consonants (plosives: p, t, k; nasals: n, m) and three different vowels (a, u, i). All items had the same syllable structure (CV.CV.CV). Minimal pairs of pseudowords were created such that they either differed only with respect to word stress (suprasegmental condition, SSEG) or only with respect to one consonant (segmental condition, SEG). There were two suprasegmental contrasts and two segmental contrasts, each consisting of two items, respectively (see Table 1). In the suprasegmental condition, penultimate stress (PU) was compared to final stress (U) and antepenultimate stress (APU) was contrasted to final stress (U). In the segmental condition, the consonants differed either in place of articulation (POA) or in a combination of place and manner of articulation (MOA). In the POA condition the consonants /m/ vs. /n/ and /k/ vs. /p/ were contrasted, whereas in the MOA condition /t/ vs. /f/ and /k/ vs. /s/ were contrasted.

**Table 1:** **Stimuli for the segmental and suprasegmental conditions**.

**Contrast**		**Item 1**	**Item 2**
Suprasegmental (SSEG)	PU vs. U	mi**pá**tu	mipa**tú**
		ta**mú**pi	tamu**pí**
	APU vs. U	**mí**kuta	miku**tá**
		**ká**timu	kati**mú**
Segmental (SEG)	POA	kúpa**mi**	kúpa**ni**
		máti**ka**	máti**pa**
	MOA	kúmi**ta**	kúmi**fa**
		tánu**ki**	tánu**si**

Contrasts are highlighted in bold face. APU, antepenultimate stress; PU, penultimate stress; U, final stress; POA, place of articulation; MOA, combination of place and manner of articulation.

For each type of stimulus, different tokens were recorded such that in each minimal pair one token was spoken by a female speaker (native speaker of Polish) and one token was spoken by a male speaker (native speaker of Persian), with the order being counterbalanced across conditions. Each pseudoword was recorded multiple times from each speaker so that different tokens from the same word were presented in the experiment. In this way, phonetic variance of stimuli was increased, disfavoring purely auditory/phonetic strategies and encouraging a more abstract, phonological type of target comparison. The duration of the pseudowords was approximately 1000 ms. Stimuli were recorded using Amadeus Pro sound editing software (HairerSoft, Kenilworth, UK).

### Pretest procedure

Each participant completed pretests to evaluate his/her basal auditory processing performance. The following three auditory cues were examined, because they are critical for word stress perception: pitch, duration and skewness. The tasks testing for pitch and length discrimination were taken from the Seashore-Test (Stanton, 1928). Skewness discrimination was determined using the procedure developed by (Haake et al., 2013). The procedure was similar to the one used in the Seashore Test. Basically, skewness discrimination required the ability to distinguish the intensity of sounds (stronger vs. weaker). All items were presented via headphones employing Adobe Audition 1.5 (Adobe Systems, San Jose, CA, USA).

Moreover, given that working memory was crucial for the sequence recall task used in the present study, measures of working memory span were determined for each participant (letter word span forward and backward, following the German version of the Wechsler Memory Scale for number word span forward and backward; Tewes, 1991). Participants were asked to repeat sequences of letters which were given by the examiner. For letter span forward, participants had first to repeat two sequences of three different letters, respectively (for example: f-b-i and c-g-e). At the second level of complexity two sequences of four different letters had to be repeated, respectively, and so forth. On the heighest (sixth) level participants had to repeat two sequences of eight letters. For the letter span backward task participants were asked to repeat two sequences of two up to eight letters, respectively, in inverted order. The test procedure was stopped when a participant repeated both sequences on a given level incorrectly.

### fMRI procedure

The experiment was a combined behavioral and fMRI study. Participants were lying in the scanner, listening to the pseudowords presented via headphones. They had response boxes in both hands and were instructed to press the correct response buttons with the index finger of the respective hand. Head movements were prevented by using soft foam pads. To familiarize participants with the task and to reduce potential training effects during fMRI data acquisition, all participants were given the opportunity to practice two blocks (one per type of contrast) in a separate room before entering the scanner. The same pseudowords as employed in the scanner served as practice items, but spoken by different speakers (a female native speaker of Dutch and a male native speaker of German).

**Figure 1:**
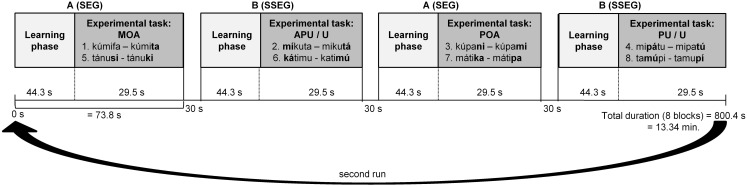
**fMRI design with 8 blocks (sequence A-B-A-B-A-B-A-B).** Each block started with a learning phase followed by the experimental task. SEG, segmental; SSEG, suprasegmental; APU, Antepenultima; MOA, Manner and place of articulation; POA, Place of articulation; PU, Penultima; U, Final syllable.

The experiment had a block design and comprised 8 blocks, each one of which lasted about 73.8 s. Each block consisted of two phases: a learning phase and an experimental phase. There were two types of blocks: Block A contained the segmental condition, and Block B the suprasegmental condition. Blocks were separated by pauses of 30 s. The blocks were presented in an alternating fashion, either starting with Block A (A-B-A-B etc.) or starting with Block B (B-A-B-A etc.), counterbalanced over participants (see Figure 1).

In each learning phase the two pseudowords needed for the following experimental task were presented, such that participants could familiarize with both words and their association with the respective response button (see Figure 1). Participants were instructed to respond to the first pseudoword encountered by pressing the right button. In this way the right button was always correct for the first pseudoword, such that no further explanation of the correct association between pseudowords and response buttons was needed. When hearing the second pseudoword of the learning phase, participants had to decide whether it matched with the first one (pressing the right button) or not (pressing the left button). Here matching refers to a phonological (type-based) rather than a phonetic (token-based) match. The participants had to make this decision in a sequence of 12 pseudowords per learning phase in pseudorandomized order such that no more than two identical items were presented in a row. The items were spoken either by the male or the female speaker, but no more than three times in a row by the same speaker. Participants were instructed to respond as fast and as accurately as possible by pressing the corresponding button after stimulus presentation. Maximum duration of response time was set to 2000 ms. Only in the learning phase Feedback was presented immediately after each trial only in the learning phase: a “Smiley” for a correct response and a “Frowney” for an incorrect or missing response. The learning phase lasted for about 44.3 s per block. At the end of the learning phase, participants had learned the correct correspondence between both pseudowords and their associated response buttons, which was also valid for the following experimental task.

In the experimental phase participants were presented with pairs of pseudowords from the set of items learned in the preceding learning phase. The task was to press the respective response buttons (as learned in the preceding learning phase) in the order the pseudowords had just been presented. No feedback was provided during the experimental phase. Eight item pairs were presented in random order per block. There were 12 different randomized orders of items for each block, such that only three to four participants had the same order of items. In each item pair, one item was spoken by the male und one by the female speaker. The duration of the experimental phase was 29.5 s per block (see Figure 1). Between pairs in the experimental phase, the background color was slightly modified (a different shade of gray for each sequence) to visually indicate the start of a new pair. Overall, the experiment took 13:34 min. The experiment was presented with Presentation software (version 14.5, Neurobehavioral Systems, Albany, USA).

### Imaging acquisition

For each participant, a high-resolution T1-weighted anatomical scan was acquired with a 3T Philips Magnetom MRI system using the standard head coil (*TR* = 9.89 s, matrix 256 × 256 mm, 176 slices, voxel size = 1 × 1 × 1 mm^3^; *FOV* = 256 mm, *TE* = 4.59 ms; flip angle = 8°). Moreover, one functional imaging block sensitive to blood oxygenation level-dependent (BOLD) contrast was recorded for each participant (T2^*^-weighted echo-planar sequence, *TR* = 2.89 s; *TE* = 30 ms; flip angle = 79°; *FOV* = 240 mm; 80 × 80 matrix; 42 slices, voxel size = 3 × 3 × 3 mm^3^, gap = 0.5 mm).

### Analysis of behavioral data

Behavioral data analysis was based responses in the experimental phase only. Furthermore, items with response latency faster than 200 ms were not considered. Analyses focused on accuracy data since reaction times in the suprasegmental condition were confounded with different “points of uniqueness” when participants were able to detect the stress difference in a pair of pseudowords (e.g., earlier point of uniqueness in “míkuta” vs. “mikúta” compared to “míkuta” vs. “mikutá”).

**Figure 2:**
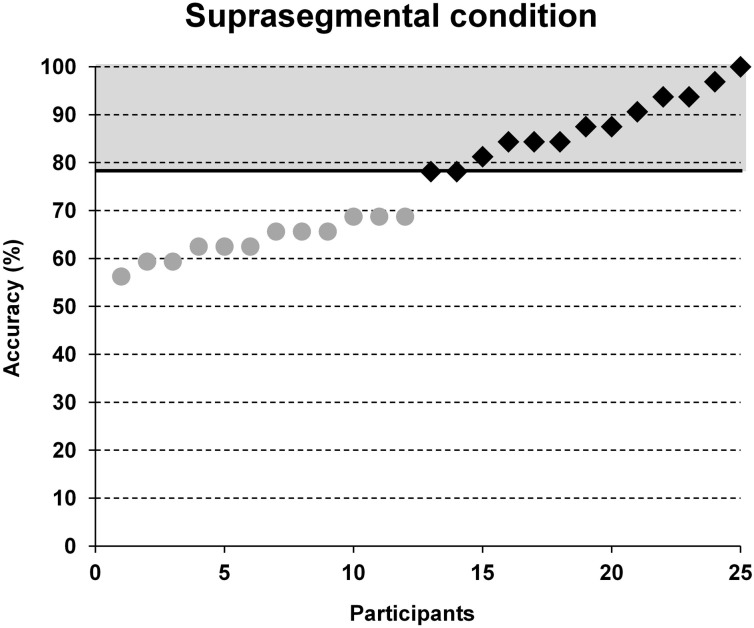
**Group classification based on a median split between accuracy results in the suprasegmental condition.** Note that chance performance would yield 50% accuracy. Black squares = participants of the above average group, gray dots = participants of the below average group.

Participant's individual performance in word stress processing was evaluated employing accuracy data of the suprasegmental condition. Based on a median split of the number of correct trials in the suprasegmental condition (see Figure 2), each participant was assigned either to a group of poor performers (below average) or to a group of good performers (above average).

In an initial step, a 2 × 2 repeated measures Analysis of Variance (ANOVA) on accuracy was performed with the within-participant factor condition (segmental vs. suprasegmental) and the between-participant factor group (above vs. below average word stress processing).

To pursue the potential association between performance in basal auditory processing, working memory, and suprasegmental processing, a stepwise multiple regression analysis with mean accuracy in the suprasegmental condition as criterion variable was conducted, which was stopped when the inclusion of another predictor would not increase R^2^ significantly (at *p* < 0.05). The predictors incorporated were performance measures from the pretest tasks, i.e., pitch discrimination, duration discrimination, skewness discrimination, a combined measure of these three auditory processing tasks (mean auditory processing accuracy), and working memory span.

### Analysis of imaging data

The anatomical scans were normalized and averaged in SPM8 (http://www.fil.ion.ucl.ac.uk/spm/software/spm8/). The fMRI time series were corrected for movement in SPM8. Images were motion corrected and realigned to each participant's first image. Data was normalized into standard MNI space. Images were resampled every 2.5 mm using 4th degree spline interpolation and smoothed with a 6 mm FWHM Gaussian kernel to accommodate inter-subject variation in brain anatomy and to increase signal to-noise ratio in the images. The data were high-pass filtered (128 s) to remove low-frequency signal drifts and corrected for autocorrelation assuming an AR(1) process. Brain activity was convolved over all experimental trials with the canonical haemodynamic response function (HRF) and its derivative.

On the first level, the intraindividual beta contrast weights for segmental and suprasegmental processing were evaluated. On the second level, both main effects and their interaction were evaluated in a 2 × 2 (flexible factorial) ANOVA with the between-subject factor group (above vs. below average) and the within-participant factor condition (segmental vs. suprasegmental). For the anatomical localization of effects, the anatomical automatic labeling tool (AAL) in SPM8 (http://www.cyceron.fr/index.php/en/plateforme-en/freeware) was used to identify Brodmann Areas (BA). If possible, the SPM Anatomy Toolbox (Eickhoff et al., 2005), available for all published cytoarchitectonic maps from www.fz-juelich.de/ime/spm_anatomy_toolbox, was additionally used and in the results will be indicated by an “Area” specification.

## Results

### Behavioral data

**Figure 3:**
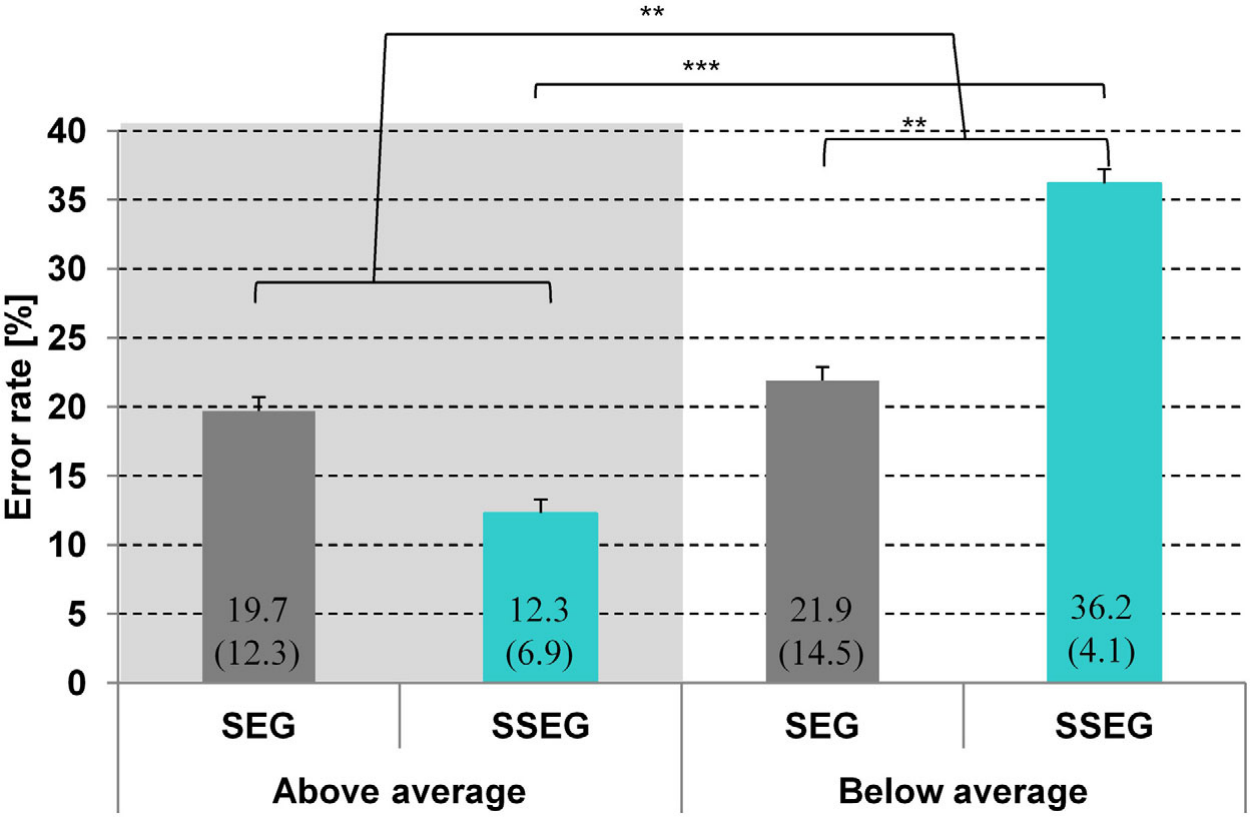
**Comparison of mean error rate (%) per condition and group.** Standard deviations are given in parentheses. SEG, segmental condition; SSEG, suprasegmental condition. ^**^p < 0.01; ^***^p < 0.001.

Accuracy in the segmental task ranged from 56.3 to 96.9% and in the suprasegmental task from 56.3 to 100%. The group classification was based on a median split for the accuracy results in the suprasegmental condition (see Figure 2). The ratio of male and female participants was comparable between both groups (good: 8m/5f, poor: 8m/4f). A descriptive overview of the results is provided in Figure 3.

A repeated measures ANOVA over arcsine-transformed error rates revealed a significant main effect of group [*F*_(1, 23)_ = 12.16, *p* < 0.01], indicating that good performers made significantly less errors (in total) than poor performers (16.0 vs. 29.0%, see Figure 3). There was no main effect of condition [*F*_(1, 23)_ < 1]. However, there was a significant two-way interaction of condition and group [*F*_(1, 23)_ = 9.3, *p* < 0.01]. The effect of condition was only significant for poor performers [*t*_(11)_ = 3.24; *p* < 0.01], meaning that in this group the error rate in the suprasegmental condition was higher than in the segmental condition (36.2 vs. 21.9%). In contrast, for good performers the effect of condition did not reach significance [*t*_(12)_ = 1.78, *p* = 0.10]. However, it should be noted that, in contrast to the poor performers, error rate was numerically higher in the segmental than in the suprasegmental condition (19.7 vs. 12.3%).

Crucially, both groups differed significantly only in the suprasegmental condition [*t*_(24)_ = 6.21, *p* < 0.001] indicating that the good performers performed reliably better (87.7%) than the poor performers (63.8%).There was no significant difference between groups for the segmental condition [*t*_(24)_ = −0.4, *p* = 0.70], see Figure 3. Furthermore, no correlation was observed between stress processing (suprasegmental) and consonant processing (segmental) (Spearman rho = 0.072, *p* = 0.733).

In order to examine whether performance in the suprasegmental condition was influenced by basic auditory processing abilities and/or working memory skills, a stepwise multiple linear regression analysis was performed over arcsine-transformed error rate of the suprasegmental condition. The final model comprised the predictors auditory processing and working memory span forward [*R*^2^ = 0.400, adjusted *R*^2^ = 0.345, *F*_(2, 24)_ = 7.3, *p* < 0.01].

### fMRI data

Analysis of fMRI data was based on all trials in the experimental phase. In a first step, a conjunction analysis was conducted to identify common overall activation in the paradigm irrespective of group and condition.

### Overview: conjunction analysis

**Figure 4:**
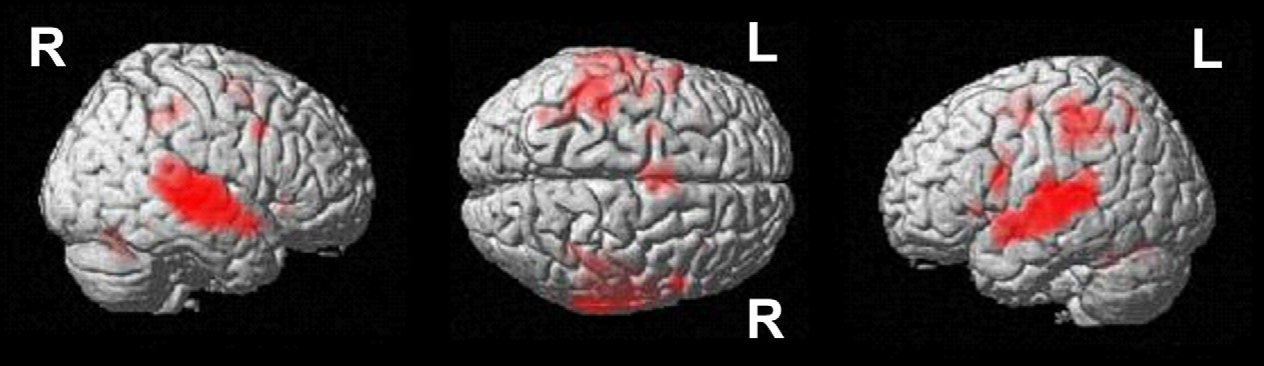
**Conjunction analysis over all groups and conditions at an uncorrected voxelwise *p* < 0.0001 (cluster-corrected FWE of *p* < 0.05)**.

A conjunction over all conditions and groups was calculated (SEG in poor performers, SSEG in poor performers, SEG in good performers, SSEG in good performers) to show joined activation at an uncorrected voxelwise *p* < 0.0001. Please note that this more rigorous *p*-value had to be used in the conjunction (compared to the level of *p* < 0.001 for the complex contrasts reported below) to visualize the different maxima of activation (cf. Wood et al., 2009; Klein et al., 2010). However, all activations reported here remain significant following family-wise error correction (FWE) at a cluster-level of *p* < 0.05. Significant activations in the entire primary auditory cortex were present (see Table 2 and Figure 4). Bilateral activation was found in the superior temporal gyrus/sulcus (STG; STS) and the middle temporal gyrus (MTG). Furthermore, left-hemispheric clusters of activated voxels were observed in the inferior frontal gyrus (IFG; Area 44, Area 6 (BA 44); SPM Anatomy Toolbox, Amunts et al., 1999; cf. Eickhoff et al., 2005), the insula, the inferior parietal sulcus (IPS; hIP2, IPC (PF, PFm), hIP1 (BA 7); SPM Anatomy Toolbox, Choi et al., 2006; cf. Eickhoff et al., 2005) the SMA, and the middle frontal gyrus (MFG). In the right hemisphere voxels in the IFG, inferior parietal lobule (hIP2, SPL (7PC), hIP1, hIP3; SPM Anatomy Toolbox, Choi et al., 2006; Scheperjans et al., 2008a,b; cf. Eickhoff et al., 2005) and the cerebellum were activated, while the precentral gyrus was found active bilaterally (see Table 2, Figure 4).

**Table 2:** **Maxima of the conjunction analysis over both conditions (segmental and suprasegmental) as well as both groups (above and below average) at an uncorrected voxelwise *p* < 0.0001 (cluster-corrected FWE of *p* < 0.05)**.

**Brain region (BA)**	**MNI**			**Cluster size**	**z score**
	***x***	***y***	***z***		
RH superior temporal gyrus/sulcus (BA 22)	57	−19	−2	963	7.62
RH middle temporal gyrus (BA 21)^a^	57	−28	−5		7.06
LH superior temporal gyrus (BA 41/42)^a^	−51	−22	4		6.52
LH middle temporal gyrus (BA 22)	−60	−31	4	885	7.13
LH insula	−33	20	1	13	4.71
LH inferior frontal gyrus (BA 44)	−57	8	16	55	5.40
RH inferior frontal gyrus (BA 47)	33	26	−5	11	4.54
LH SMA (BA 6)	−3	−4	58	82	4.57
LH IPS (BA 7)	−45	−43	43	293	5.30
RH IPS (BA 7)	39	−43	43	41	4.84
LH precentral gyrus (BA 6)	−48	−4	46	11	4.56
RH precentral gyrus (BA 6)	57	11	37	17	4.49
LH middle frontal gyrus (BA 6)	−27	−4	52	17	4.37
RH cerebellum	6	−67	−20	58	4.82

IPS, inferior parietal sulcus; LH, left hemisphere; RH, right hemisphere; SMA, supplementary motor area.

a Minor maximum.

### Condition-based comparisons

#### Suprasegmental vs. segmental processing

**Figure 5:**
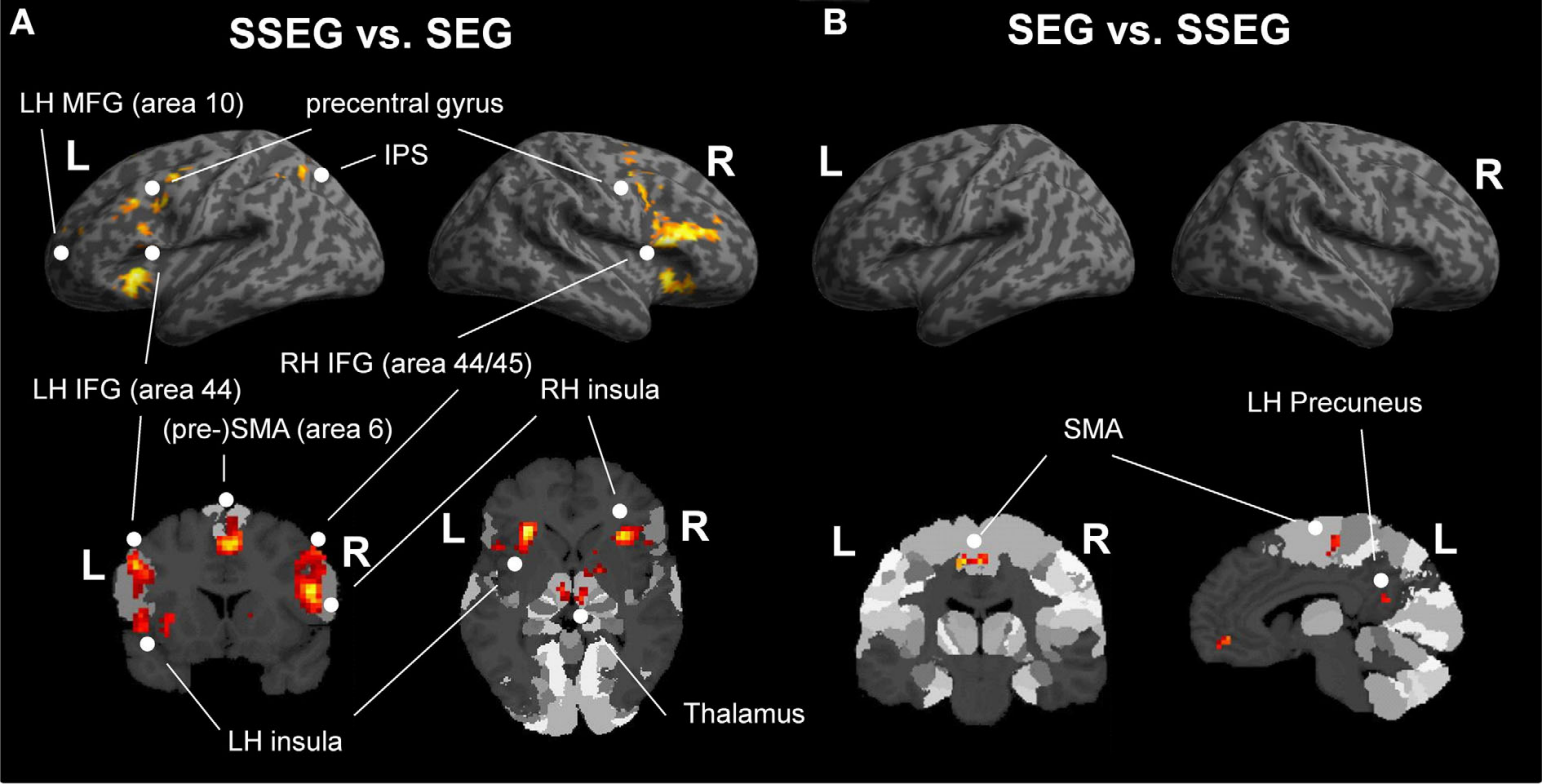
**(A)** Comparison of suprasegmental vs. segmental condition (uncorrected *p* < 0.001, *k* = 10 voxels). **(B)** Segmental vs. suprasegmental condition (uncorrected *p* < 0.001, *k* = 10 voxels). IFG, inferior frontal gyrus; IPS, inferior parietal sulcus; LH, left hemisphere; MFG, middle frontal gyrus; RH, right hemisphere; SEG, segmental; SMA, supplementary motor area; SSEG, suprasegmental.

Suprasegmental was contrasted to segmental processing at an uncorrected voxelwise threshold of *p* < 0.001 and a cluster size of *k* = 10 voxels (see Figure 5A, Table 3). Larger activation for suprasegmental processing was found bilaterally in the IFG (Area 44 and Area 45 (BA44 and BA 45); SPM Anatomy Toolbox, cf. Eickhoff et al., 2005) as well as in the insula. Furthermore, in the left hemisphere the thalamus, the IPS (hIP1, hIP3 (BA 7); SPM Anatomy Toolbox, cf. Eickhoff et al., 2005) and the pre-SMA (BA 6) were activated, while in the right hemisphere the pallidum as well as the right SMA (BA 6) revealed stronger activation in stress processing compared to consonant processing. Further clusters of activated voxels were found in the bilateral precentral gyrus, in the left MFG (BA 10) and in the cerebellum, bilaterally.

**Table 3:** **Significant brain activation differences for various group and condition contrasts**.

**Contrast**	**Brain region (BA)**	**MNI**			**Cluster size**	**z score**
		***x***	***y***	***z***		
Suprasegmental vs. segmental	RH inferior frontal gyrus (BA 44/45)	51	17	16	345	5.19
	RH inferior frontal gyrus (BA 45)^a^	54	29	22		4.97
	LH inferior frontal gyrus (BA 44/45)	−51	14	31	112	4.38
	LH insula	−30	20	−11	115	5.15
	RH insula	33	23	−2	134	4.50
	LH thalamus	−6	−13	−2	151	3.85
	RH pallidum	18	−1	1	70	4.02
	LH IPS [hIP1, hIP3 (BA 7)]	−36	−52	37	79	4.08
	LH pre−SMA (BA 6)	0	17	46	184	4.95
	RH SMA (BA 6)	9	2	58	12	3.67
	RH precentral gyrus (BA 6)	42	−1	49	100	4.37
	LH precentral gyrus (BA 6)	−39	−4	40	55	4.31
	LH middle frontal gyrus (BA 10)	−30	53	22	16	3.94
	LH cerebellum	−9	−76	−29	140	4.05
	RH cerebellum	24	−34	−41	31	4.01
Segmental vs. suprasegmental	LH SMA (BA 6)	−9	−19	52	44	3.98
	RH SMA (BA 6)	15	−10	40	19	4.09
	RH middle orbital gyrus (BA 10)	3	50	−5	88	4.19
	LH precuneus (BA 7)	−6	−58	19	13	3.47
Below vs. above	LH middle temporal gyrus (BA 21)	−69	−34	−8	16	3.84
Above vs. below	LH precunes (BA 7)	0	−52	40	24	3.71
Interaction group ^*^ condition	RH hippocampus	30	−34	−2	11	3.76
	RH cerebellum	24	−31	−23	16	4.48

IPS, inferior parietal sulcus; LH, left hemisphere; RH, right hemisphere; SMA, supplementary motor area.

a Minor maximum.

#### Segmental vs. suprasegmental processing

Inspection of the inverse contrast (uncorrected *p* < 0.001, *k* = 10 voxel) revealed activation in the bilateral SMA (BA 6), the right middle orbital gyrus and the left precuneus (see Figure 5B, Table 3).

### Group-based comparisons

#### Poor performers vs. good performers

**Figure 6:**
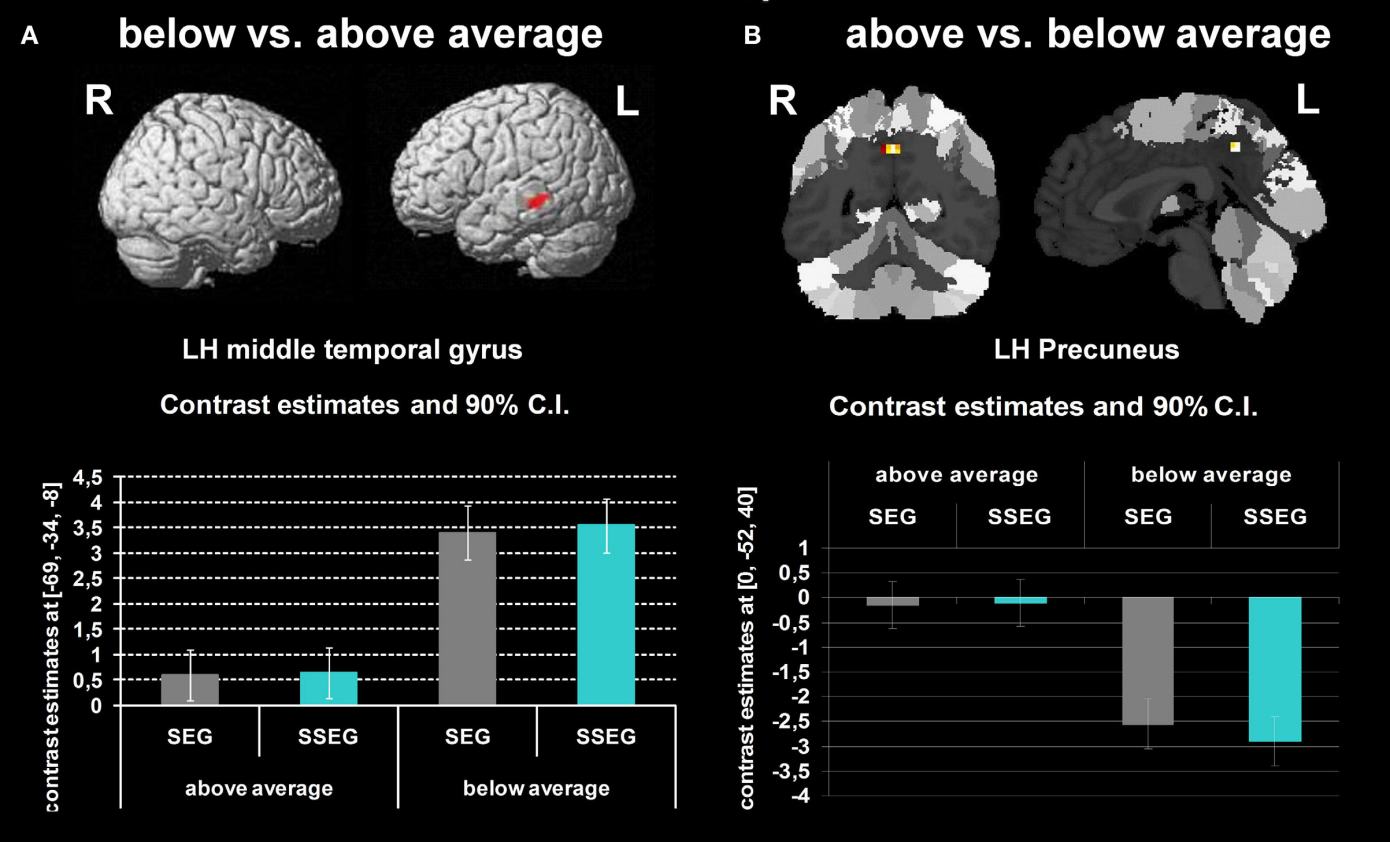
**(A)** Comparison of participants below vs. above average (uncorrected *p* < 0.001, *k* = 10 voxels). **(B)** Participants above vs. below average (uncorrected *p* < 0.001, *k* = 10 voxels). The bar charts below the activation figure depict the corresponding beta estimates for the respective brain region.

Poor performers revealed significantly stronger activation than good performers in the left MTG at an uncorrected voxelwise *p* < 0.001 and a cluster size of 10 voxels (see Figure 6A, Table 3).

#### Good performers vs. poor performers

When comparing good performers vs. poor performers (uncorrected *p* < 0.001, *k* = 10 voxel), significantly more activation was found in the left precuneus (see Figure 6B, Table 3).

### Interaction between group and condition

We conducted an ANCOVA over participants on the fMRI data with working memory and auditory performance from the pretest as covariates, to correct the segmental and suprasegmental activations for working memory and auditory abilities. In this context, we also examined whether there is additional fMRI variance, which is exclusively explained by the covariates. However, at the threshold given (FWE-cluster threshold corrected) there was no such additional activity to be found.

**Figure 7:**
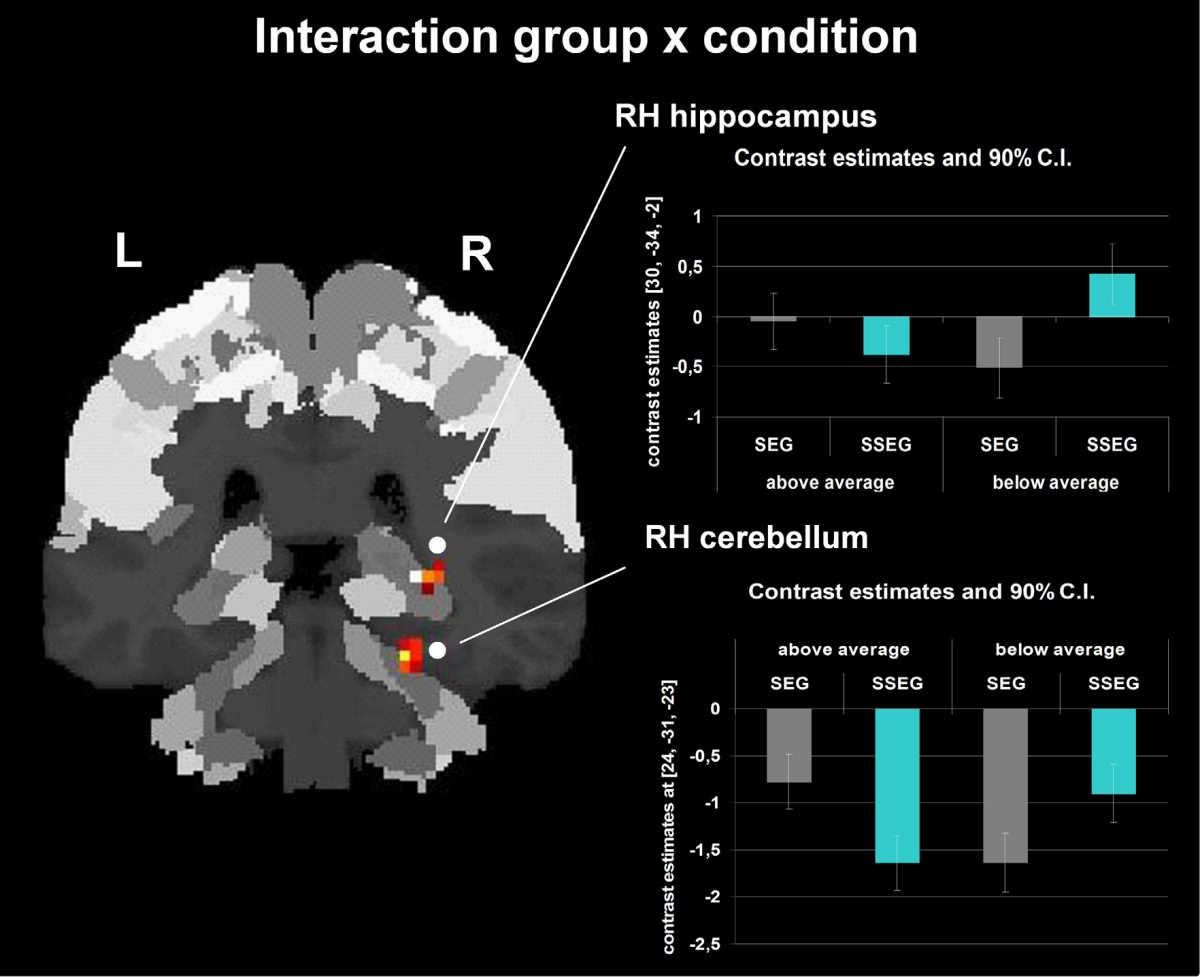
**Interaction between group and condition (uncorrected *p* < 0.001, *k* = 10 voxels).** The bar charts next to the activation figure depict the corresponding beta estimates for the respective brain region.

Group and condition interacted significantly in the right hippocampus (CA (BA 27), SPM Anatomy Toolbox, Amunts et al., 2005; cf. Eickhoff et al., 2005) and cerebellum at an uncorrected voxelwise *p* < 0.001 and a cluster size of 10 voxels (see Figure 7, Table 3). However, especially in the cerebellum the interactions in signal change seem to be mostly due to different degrees of deactivation. However, it can be seen that good performers showed relatively more activation (or less deactivation, respectively) in the segmental condition in the right hippocampus and cerebellum compared to poor performers, whereas poor performers revealed relatively stronger activation compared to good performers in these areas in the suprasegmental condition.

## Discussion

The current study set off to examine whether there are interindividual differences in word stress processing performance in native speakers of German and, if so, which neural correlates underlie these differences. So far, most studies focused on typologically motivated processing differences between speakers of languages with fixed vs. variable stress. In particular, Dupoux, Peperkamp and colleagues compared speakers of Spanish (variable stress pattern) to speakers of French (fixed stress pattern; see Dupoux et al., 1997, 2001; Peperkamp et al., 1999; Peperkamp and Dupoux, 2002) and found superior performance of the former compared to the latter (for similar results in a comparison between French and German see Schmidt-Kassow et al., 2011a). Interindividual differences within one language—although repeatedly observed—were treated as noise (Peperkamp et al., 1999; Domahs et al., 2008, 2013b; Dupoux et al., 2010) or were left unexplained (Boecker et al., 1999).

In the present study, participants were examined in both suprasegmental as well as segmental variants of the sequence recall task both at a behavioral and at a neuro-functional level. Indeed, based on behavioral results we were able to identify considerable interindividual differences within native speakers of German (accuracy in the suprasegmental task ranging from floor to ceiling performance).

To explore more thoroughly, which factors modulate suprasegmental processing differences, working memory span as well as auditory processing abilities were analyzed. In fact, we demonstrated that suprasegmental performance was predicted by both basic auditory processing abilities (i.e., duration, time, skewness discrimination) and working memory span. The influence of working memory on performance in the suprasegmental task seems highly plausible since working memory was clearly task-relevant. Crucially, the fact that a combined measure of duration, time, and skewness discrimination predicted individual performance in word stress processing, provides a first hint toward an explanation for the interindividual variability observed. This result fits nicely with findings recently reported by Haake et al. (2013), who observed that word stress processing in children with specific language impairment as well as in typically developing children is predicted by auditory processing of duration cues. Obviously, basic auditory processing performance may exert its influence not only in children, but also in healthy adults for whom the recognition and interpretation of word stress is relevant in their native language.

In sum, there was substantial interindividual variability in word stress processing. Hence, two groups were defined based on a median split of individual accuracy results in the suprasegmental task. Neural correlates of segmental and suprasegmental processing and their interaction with group membership were investigated and will be discussed in the following.

### Neural correlates of segmental and suprasegmental processing

The conjunction analysis revealed a large cluster of activation in auditory cortex across performance levels and conditions (cf. Figure 4, Table 2), extending from the superior temporal gyrus to the middle temporal gyrus and to the insula. This finding is highly plausible, because participants had to process auditory linguistic stimuli. More specifically, previous studies reported activation in the STG or STS for processing of prosodic information in general (e.g., Dogil, 2003; Ischebeck et al., 2008), and for processing of word stress in particular (Aleman et al., 2005; Klein et al., 2011; Domahs et al., 2013b).

In addition, activation in the bilateral supplementary motor area (with left-hemispheric peak activation within a large cluster extending into the right hemisphere) and in the bilateral inferior parietal sulcus was found. This may be related to the fact that participants had to determine either stress localization or consonant differences by button presses since the SMA has been suggested to subserve decision making (Kong et al., 2005). Additionally, a combination of working memory related BA 44 and intraparietal BA 7 activation indicated that participants had to hold the sequences of pseudowords in working memory. Moreover, bilateral activation in the precentral gyrus was observed, probably indicating motor processing associated with finger movements and button presses (Zilles and Rehkämper, 1998).

Beyond these task-related effects, cerebellum, temporal cortex, premotor cortex, preSMA/SMA and inferior frontal cortex have been described as part of a network involved in speech perception, especially engaged in the temporal processing of speech (Grahn and Brett, 2007; Kotz et al., 2009; Kotz and Schwartze, 2010).

### Suprasegmental vs. segmental processing

In the behavioral data, no correlation was observed between stress processing (suprasegmental) and consonant processing (segmental). This suggests that the linguistic abilities underlying these two conditions may be to a certain degree independent, although they were tested with a comparable paradigm in the present study.

When the suprasegmental task was contrasted to the segmental task, a subcortico-cortico-cerebellar network of brain regions was revealed, including bilateral IFG (BA44 and BA 45), bilateral insula, bilateral precentral gyrus, bilateral cerebellum, left thalamus, left pre-SMA (BA 6), right globus pallidus, and right SMA (BA 6). There is accumulating evidence, that this network is involved in processing spectro-temporal aspects of speech (Lutz et al., 2000; Lewis et al., 2004; Bengtsson et al., 2005; Riecker et al., 2006; Grahn and Brett, 2007; Coull et al., 2008; Geiser et al., 2008; Kotz et al., 2009; Kotz and Schwartze, 2011; Schwartze et al., 2012a,b, see Kotz and Schwartze, 2010, for a review). This finding seems very plausible, given that duration is the most relevant acoustic cue to word stress in German (Jessen and Marasek, 1997; Classen et al., 1998; Schneider, 2007; Schneider and Möbius, 2007; Lintfert, 2010) and performance in auditory discrimination in general and duration discrimination in particular predicts performance in the more complex task related to word stress (behavioral results of the present study, see Haake et al., 2013, for evidence from German speaking children).

More specifically, bilateral activation in the inferior frontal gyri related to the suprasegmental condition is in line with previous studies, which reported these areas to be activated in processing linguistic aspects of prosody (e.g., Wildgruber et al., 2004; Li et al., 2010; Klein et al., 2011; Domahs et al., 2013b).

Furthermore, activation in the left insula related to suprasegmental processing is consistent with previous studies, which found this area activated for auditory temporal processing (Lewis et al., 2000; Ackermann et al., 2001; Lewis and Miall, 2003), for pitch-related stimuli (Zarate and Zatorre, 2005) as well as for auditory timing perception (Geiser et al., 2008) and word stress processing proper (Aleman et al., 2005; Klein et al., 2011).

Activation in the bilateral inferior parietal sulcus may reflect the fact that participants had to store information in working memory and to respond by button presses. Possibly, they employed a spatial representation of the pseudowords (e.g., first syllable = left, last syllable = right) and of response buttons to come to the correct decision. Amongst others, the intraparietal cortex has been suggested to subserve mental imagery (Just et al., 2004). Moreover, the IPS has been frequently reported to be involved in the processing of proximity relations (see Dehaene et al., 2003 for a review). Recall that stress is an inherently relational property and requires the comparison of acoustic cues (e.g., duration, pitch, and skewness) between stressed and unstressed syllables. In the present study, the inferior parietal sulcus may be associated with mental imagery and with the evaluation of gradual differences in acoustic cues related to word stress. This might comprise positional information, which has to be encoded in the IPS and held in working memory as well as the actual comparison process of the positional information within the sequences of CV-syllables—a process also most probably associated with the intraparietal cortices (cf. Klein et al., 2011). In particular, bilateral inferior parietal cortex has been found activated in tasks tapping on suprasegmental compared to segmental aspects of words (Li et al., 2010; Klein et al., 2011).

Beyond temporal processing of speech input, activation in the supplementary motor area may be related to the fact that in general the suprasegmental task in this study was somewhat more difficult than the segmental task. The SMA has been found to support operation procedures (Kong et al., 2005). Interestingly, Domahs et al. (2013b) observed increased activation in bilateral SMA in a difficult compared to an easy condition in a word stress violation task. Moreover, SMA activation in the suprasegmental condition together with a significantly increased activation in the precentral gyrus could point to an involvement of the central motor system. Given that both the SMA and the precentral gyrus were activated bilaterally, these findings may reflect control of finger movements in participants (e.g., Shibasaki et al., 1993; Catalan et al., 1998). Possibly, participants may have needed higher control of their finger movements in the more difficult suprasegmental condition. An alternative explanation could be that in more difficult conditions participants may establish a correspondence between their fingers and the positional information of stress, for instance, by using finger counting. This would be also in line with the activation pattern observed in SMA, precentral and intraparietal areas. However, this account remains speculative so far and needs further evaluation in future studies.

### Interindividual differences

The middle temporal gyrus was found activated in both conditions (segmental, suprasegmental) for both groups (cf. Table 3). This fits well with the fact that the MTG has been associated with phonology (Graves et al., 2010) and, more generally, with complex sound and speech processing (Scott et al., 2000). Nevertheless, poor performers showed stronger activation in this region.

Further significant changes in the BOLD signal were found in the precuneus. These findings are rather difficult to interpret since for good performers the BOLD signal in the precuneus seemed to be close to zero in both the segmental and the suprasegmental conditions (see Figure 6B), whereas in poor performers the precuneus was strongly deactivated in both conditions. Considering that the amplitude of the BOLD signal indicated by SPM is subject to arbitrary factors (such as the definition of the baseline), the present findings can only be interpreted in relative terms, not in terms of “activation” or “deactivation.” Generally, the precuneus has been suggested not only to subserve learning of motor-sequences (Sadato et al., 1996; Sakai et al., 1998) but also to be involved in mental imagery (Dehaene et al., 1996; Huijbers et al., 2011). Possibly, good performers may have relied more on mental imagery or motor-sequence learning to solve the task correctly, compared to poor performers. Nevertheless, we are well aware of the fact that currently this explanation remains speculative.

One may conclude that both groups activated the MTG for phonological processing of stimuli in both conditions, but that poor performers required more resources. It may be speculated that good performers have used a combination of visual and auditory representations to solve the tasks, whereas poor performers only relied on auditory information (but to a higher degree). Possibly, a combination of visual and auditory processing may be advantageous.

Although native speakers of German are highly familiar with the use of suprasegmental features in their mother tongue, the present study shows that their performance in an experimental task tapping on this aspect of language may nevertheless be very heterogeneous. Until now, it was assumed that native German speakers should be “naturally” competent in word stress processing, since this is a relevant feature of their language, which is acquired early. Preverbal infants learn the typical stress pattern of their mother tongue and can use it in speech segmentation (Hoehle et al., 2009). Importantly, even those participants, who showed poor performance in the specific suprasegmental task in the present study, were competent speakers of German. Note that the stress pattern of real words is stored in the lexicon. However, in the present study, participants had to process pseudowords which by definition cannot be stored in the mental lexicon. Thus, processing word stress in everyday language requires lexical retrieval, whereas the suprasegmental task in our experiment may have required other types of prosodic knowledge (e.g., rule-based knowledge). Furthermore, every-day language is typically embedded in a redundant context, which helps in resolving ambiguities related to word stress, e.g., in the interpretation of minimal pairs. Therefore, the specific difficulties in suprasegmental processing of pseudowords observed in the present study are subclinical with no obvious impact on language use.

### Interaction between group and condition

Behaviorally, a two-way interaction of condition (segmental vs. suprasegmental) and group (below vs. above average) indicated that the good performers were numerically better in suprasegmental than in segmental processing, whereas the poor performers were significantly better in segmental than in suprasegmental processing (see Figure 3). Importantly, a two-way interaction of condition and group was also revealed in the neuro-functional data (see Figure 7, Table 3). Good performers showed relatively more activation (or less deactivation, respectively) in the segmental condition in the right hippocampus and cerebellum compared to poor performers, whereas poor performers revealed relatively stronger activation in these areas in the suprasegmental condition compared to good performers.

Hippocampal cells have been shown to be involved in auditory working memory in rats (Sakurai, 1990, 1994). More recently, the hippocampus has been argued to contribute to performance in a variety of cognitive tasks including working memory and perception, when these tasks require high-resolution binding of features and relational information (Yonelinas, 2013). Clearly, the sequence recall task used in the present experiment does require such a complex and demanding type of binding. Interestingly, activation in the right hippocampus was related to relative task difficulty: Poor performers seemed to need relatively more cognitive resources in the suprasegmental task (which they performed worse than the segmental task), but good performers seemed to put relatively more effort into the segmental task (which they performed worse than the suprasegmental task).

Furthermore, a similar pattern of (de-)activation was observed for the interaction in the right cerebellum. The cerebellum has been considered to be part of a network related to the processing of spectro-temporal aspects of speech (Kotz and Schwartze, 2010). The interaction in the cerebellum suggests that poor performers may have needed the cerebellum relatively more for the suprasegmental task (although achieving inferior results) than good performers. The opposite pattern was observed in the segmental condition. Again, these interpretations have to be considered very cautiously and remain speculative, because the interaction pattern consists only of different degrees of deactivation.

## Conclusion and perspectives

The present study is a first step toward a more comprehensive understanding of the processing of word stress. In particular, it highlights the need to examine brain activation data not only at the second level in group analyses, but also to analyze individual data at the first level. Taken together, our results provide behavioral and neuro-functional evidence for substantial interindividual differences within a group of native speakers of German, a language with variable stress, in word stress processing. They suggest that part of the behavioral variance is explained by basic auditory processing and working memory performance. It would be interesting to explore, whether speakers of a language with fixed stress (e.g., Czech, Finnish, Polish, Turkish, Persian, or French) show similar interindividual heterogeneity.

## Conflict of interest statement

The authors declare that the research was conducted in the absence of any commercial or financial relationships that could be construed as a potential conflict of interest.

## References

[B1] Ackermann H. Riecker A. Mathiak K. Erb M. Grodd W. Wildgruber D. (2001). Rate-dependent activation of a prefrontal–insular–cerebellar network during passive listening to trains of click stimuli: an fMRI study. Neuroreport 12, 4087–4092 10.1097/00001756-200112210-00045 11742243

[B2] Aleman A. Formisano E. Koppenhagen H. Hagoort P. de Haan E. H. F. Kahn K. (2005). The functional neuroanatomy of metrical stress evaluation of perceived and imagined spoken words. Cereb. Cortex 15, 221–228 10.1093/cercor/bhh124 15269107

[B3] Amunts K. Kedo O. Kindler M. Pieperhoff P. Mohlberg H. Shah N. J. (2005). Cytoarchitectonic mapping of the human amygdala, hippocampal region and entorhinal cortex: intersubject variability and probability maps. Anat. Embryol. 210, 343–352 10.1007/s00429-005-0025-5 16208455

[B4] Amunts K. Schleicher A. Burgel U. Mohlberg H. Uylings H. B. Zilles K. (1999). Broca's region revisited: cytoarchitecture and intersubject variability. J. Comp. Neurol. 412, 319–341 10441759 10.1002/(sici)1096-9861(19990920)412:2<319::aid-cne10>3.0.co;2-7

[B5] Bengtsson S. L. Ehrsson H. H. Forssberg H. Ullen F. (2005). Effector-independent voluntary timing: behavioral and neuroimaging evidence. Eur. J. Neurosci. 22, 3255–3265 10.1111/j.1460-9568.2005.04517.x 16367791

[B6] Boecker K. B. E. Bastiaansen M. C. M. Vroomen J. Brunia C. H. M. De Gelder B. (1999). An ERP correlate of metrical stress in spoken word recognition. Psychophysiology 36, 706–720 10.1111/1469-8986.3660706 10554585

[B7] Catalan M. J. Honda M. Weeks R. A. Cohen L. G. Hallett M. (1998). The functional neuroanatomy of simple and complex sequential finger movements: a PET study. Brain 121(Pt 2), 253–264 10.1093/brain/121.2.253 9549504

[B8] Choi H. J. Zilles K. Mohlberg H. Schleicher Al. Fink G. R. Armstrong E. (2006). Cytoarchitectonic identification and probabilistic mapping oft wo distinct areas within the anterior ventral bank of the human intraparietal sulcus. J. Comp. Neurol. 495, 53–69 10.1002/cne.20849 16432904 PMC3429851

[B9] Classen K. Dogil G. Jessen M. Marasek K. Wokurek W. (1998). Stimmqualität und Wortbetonung im Deutschen. Linguist. Berichte 174, 202–245

[B10] Coull J. T. Nazarian B. Vidal F. (2008). Timing, storage, and comparison of stimulus duration engage discrete anatomical components of a perceptual timing network. J. Cogn. Neurosci. 20, 2185–2197 10.1162/jocn.2008.20153 18457512

[B11] Dehaene S. Piazza M. Pinel P. Cohen L. (2003). Three parietal circuits for number processing. Cogn. Neuropsychol. 20, 487–506 10.1080/02643290244000239 20957581

[B12] Dehaene S. Tzourio N. Frak V. Raynaud L. Cohen L. Mehler J. (1996). Cerebral activations during number multiplication and comparison: a PET study. Neuropsychologia 34, 1097–1106 10.1016/0028-3932(96)00027-9 8904747

[B13] Dogil G. (2003). Understanding prosody, in Psycholinguistik: Ein Internationales Handbuch, eds Rickheit H. Herrmann T. Deutsch W. (Berlin: de Gruyter), 544–565

[B14] Domahs U. Genc S. Knaus J. Wiese R. Kabak B. (2013a). Processing (un-)predictable word stress: ERP evidence from Turkish. Lang. Cogn. Process. 28, 335–354 10.1080/01690965.2011.634590

[B15] Domahs U. Klein E. Huber W. Domahs F. (2013b). Good, bad and ugly word stress – fMRI evidence for foot structure driven processing of prosodic violations. Brain Lang. 125, 272–282 10.1016/j.bandl.2013.02.012 23587493

[B16] Domahs U. Knaus J. Orzechowska P. Wiese R. (2012). Stress “deafness” in a language with fixed word stress: an ERP study on Polish. Front. Lang. Sci. 3:439 10.3389/fpsyg.2012.00439 23125839 PMC3485581

[B17] Domahs U. Wiese R. Bornkessel-Schlesewsky I. Schlesewsky M. (2008). The processing of German word stress: evidence for the prosodic hierarchy. Phonology 25, 1–36 10.1017/S0952675708001383

[B18] Dupoux E. Pallier C. Sebastian N. Mehler J. (1997). A destressing “Deafness“ in French? J. Mem. Lang. 36, 406–421 10.1006/jmla.1996.250017592731

[B19] Dupoux E. Peperkamp S. Sebastián-Gallés N. (2001). A robust method to study stress “deafness.” J. Acoust. Soc. Am. 110, 1606–1618 10.1121/1.1380437 11572370

[B20] Dupoux E. Peperkamp S. Sebastián-Gallés N. (2010). Limits on bilingualism revisited: stress ‘deafness’ in simultaneous French-Spanish bilinguals. Cognition 114, 266–275 10.1016/j.cognition.2009.10.001 19896647

[B21] Dupoux E. Sebastián-Gallés N. Navarrete E. Peperkamp S. (2008). Persistent stress ‘deafness’: the case of French learners of Spanish. Cognition 106, 682–706 10.1016/j.cognition.2007.04.001 17592731

[B22] Eickhoff S. Stephan K. E. Mohlberg H. Grefkes C. Fink G. R. Amunts K. (2005). A new SPM toolbox for combining probabilistic cytoarchitectonic maps and functional imaging data. Neuroimage 25, 1325–1335 10.1016/j.neuroimage.2004.12.034 15850749

[B23] Eisenberg P. (1991). Syllabische Struktur und Wortakzent: Prinzipien der Prosodik deutscher Wörter. Zeitschrift für Sprachwissenschaft, 10, 37–64 10.1515/zfsw.1991.10.1.37

[B24] Eisenberg P. (2006). Grundriss der deutschen Grammatik: Das Wort, 3rd Edn. Stuttgart: Metzler

[B25] Féry C. (1998). German word stress in optimality theory. J. Comp. Ger. Linguist. 2, 101–142 10.1023/A:1009883701003

[B26] Geiser E. Zaehle T. Jancke L. Meyer M. (2008). The neural correlate of speech rhythm as evidenced by meter processing: an fMRI study. J. Cogn. Neurosci. 20, 541–552 10.1162/jocn.2008.20029 18004944

[B27] Giegerich H. J. (1985). Metrical Phonology and Phonological Structure: German and English. Cambridge: Cambridge University Press

[B28] Grahn J. A. Brett M. (2007). Rhythm and beat perception in motor areas of the brain. J. Cogn. Neurosci. 19, 893–906 10.1162/jocn.2007.19.5.893 17488212

[B29] Graves W. W. Desai R. Humphries C. Seidenberg M. S. Binder J. R. (2010). Neural systems for reading aloud: a multiparametric approach. Cereb. Cortex 20, 1799–1815 10.1093/cercor/bhp245 19920057 PMC2901017

[B30] Haake C. Kob M. Willmes K. Domahs F. (2013). Word stress processing in specific language impairment: auditory or representational deficits? Clin. Linguist. Phon. 27, 594–615 10.3109/02699206.2013.798034 23806129

[B31] Heim S. Alter K. (2006). Prosodic pitch accents in language comprehension and production: ERP data and acoustic analyses. Acta Neurobiol. Exp. 66, 55–68 16617677 10.55782/ane-2006-1587

[B32] Heim S. Alter K. (2007). Focus on focus: the brain's electrophysiological response to focus particles and accents in German, in Interface and Interface Conditions. Language, Context and Cognition, ed Späth A. (Berlin; New York: de Gruyter), 277–297

[B33] Hoehle B. Bijeljac-Babic R. Herold B. Weissenborn J. Nazzi T. (2009). Language specific prosodic preferences during the first half year of life: evidence from German and French infants. Infant Behav. Dev. 32, 262–274 10.1016/j.infbeh.2009.03.004 19427039

[B34] Huijbers W. Pennartz C. M. A. Rubin D. C. Daselaar S. M. (2011). Imagery and retrieval of auditory and visual information: neural correlates of successful and unsuccessful performance. Neuropsychologia 49, 1730–1740 10.1016/j.neuropsychologia.2011.02.051 21396384

[B35] Ischebeck A. Friederici A. D. Alter K. (2008). Processing prosodic boundaries in natural and hummed speech. An fMRI study. Cereb. Cortex 18, 541–552 10.1093/cercor/bhm083 17591598

[B36] Janssen U. Domahs F. (2008). Going on with optimized feet: evidence for the interaction between segmental and metrical structure from a case of Primary Progressive Aphasia. Aphasiology 22, 1157–1175 10.1080/02687030701820436

[B37] Jessen M. Marasek K. (1997). Voice quality correlates of word stress in tense versus lax vowels in German, in Larynx (Marseille), 127–130

[B38] Just M. A. Newman S. D. Keller T. A. McEleny A. Carpenter P. (2004). Imagery insentence comprehension: an fMRI study. Neuroimage 21, 112–124 10.1016/j.neuroimage.2003.08.042 14741648

[B39] Kaltenbacher E. (1994). Typologische Aspekte des Wortakzents: Zum Zusammenhang von Akzentposition und Silbengewicht im Arabischen und im Deutschen. Zeitschrift für Sprachwissenschaft 13, 20–55 10.1515/zfsw.1994.13.1.20

[B40] Klein E. Domahs U. Grande M. Domahs F. (2011). Neuro-cognitive foundations of word stress processing—evidence from fMRT. Behav. Brain Funct. 7:15 10.1186/1744-9081-7-15 21575209 PMC3120660

[B41] Klein E. Willmes K. Dressel K. Domahs F. Wood G. Nuerk H.-C. (2010). Categorial and continuous—disentangling the neural correlates of the carry effect in multi-digit addition. Behav. Brain Funct. 6:70 10.1186/1744-9081-6-70 21092129 PMC3002291

[B42] Knaus J. Domahs U. (2009). Experimental evidence for optimal and minimal metrical structure of German word prosody. Lingua 119, 1396–1413 10.1016/j.lingua.2008.04.002

[B43] Kong J. Wang C. Kwong K. Vangel M. Chuac E. Gollub R. (2005). The neural substrate of arithmetic operations and procedure complexity. Cogn. Brain Res. 22, 397–405 10.1016/j.cogbrainres.2004.09.011 15722210

[B44] Kotz S. A. Schwartze M. (2010). Cortical speech processing unplugged: a timely subcortico-cortical framework. Trends Cogn. Sci. 14, 392–399 10.1016/j.tics.2010.06.005 20655802

[B45] Kotz S. A. Schwartze M. (2011). Differential input of the supplementary motor area to a dedicated temporal processing network: functional and clinical implications. Front. Integr. Neurosci. 5, 1–4 10.3389/fnint.2011.00086 22363269 PMC3277277

[B46] Kotz S. A. Schwartze M. Schmidt-Kassow M. (2009). Non-motor basal ganglia functions: a review and proposal for a model of sensory predictability in auditory language perception. Cortex 45, 982–990 10.1016/j.cortex.2009.02.010 19361785

[B47] Levelt W. J. M. Roelofs A. Meyer A. S. (1999). A theory of lexical access in speech production. Behav. Brain Sci. 22, 1–75 10.1017/S0140525X99001776 11301520

[B48] Lewis J. W. Beauchamp M. S. DeYoe E. A. (2000). A comparison of visual and auditory motion processing in human cerebral cortex. Cereb. Cortex 10, 873–888 10.1093/cercor/10.9.873 10982748

[B49] Lewis P. A. Miall R. C. (2003). Brain activation patterns during measurement of sub- and supra-second intervals. Neuropsychologia 41, 1583–1592 10.1016/S0028-3932(03)00118-0 12887983

[B50] Lewis P. A. Wing A. M. Pope P. A. Praamstra P. Miall R. C. (2004). Brain activity correlates differentially with increasing temporal complexity of rhythms during initialisation, synchronisation, and continuation phases of paced finger tapping. Neuropsychologia 42, 1301–1312 10.1016/j.neuropsychologia.2004.03.001 15193939

[B51] Li X. Gandour J. T. Talavage T. Wong D. Hoffa A. Lowe M. (2010). Hemispheric asymmetries in phonological processing of tones versus segmental units. Neuroreport 21, 690–694 10.1097/WNR.0b013e32833b0a10 20508544 PMC2890240

[B52] Lintfert B. (2010). Phonetic and Phonological Development of Stress in German. Doctoral thesis, Universität Stuttgart, Stuttgart.

[B53] Lutz K. Specht K. Shah N. J. Jancke L. (2000). Tapping movements according to regular and irregular visual timing signals investigated with fMRI. Neuroreport 11, 1301–1306 10.1097/00001756-200004270-00031 10817611

[B54] Mehler J. Dupoux E. Pallier C. Dehaene-Lambertz G. (2004). Cross-linguistic approaches to speech processing. Curr. Opin. Neurobiol. 4, 171–176 10.1016/0959-4388(94)90068-X 8038572

[B55] Molczanow J. Domahs U. Knaus J. Wiese R. (2013). The lexical representation of word stress in Russian: evidence from event-related potentials. Ment. Lex. 8, 164–194 10.1075/ml.8.2.03mol

[B56] Peperkamp S. Dupoux E. (2002). “A typological study of stress “deafness,” in Laboratory Phonology 7, eds Gussenhoven C. Warner N. (Berlin: Walter de Gruyter GmbH & Co. KG), 203–240

[B57] Peperkamp S. Dupoux E. Sebastián-Gallés N. (1999). Perception of stress by French, Spanish, and bilingual subjects, in ESCA 7th European Conference on Speech Communication and Technology (Budapest).

[B58] Peperkamp S. Vendelin I. Dupoux E. (2010). Perception of predictable stress: a cross-lingusitic investigation. J. Phon. 38, 422–430 10.1016/j.wocn.2010.04.001

[B59] Riecker A. Kassubek J. Gröschel K. Grodd W. Ackermann H. (2006). The cerebral control of speech tempo: opposite relationship between speaking rate and BOLD signal changes at striatal and cerebellar structures. Neuroimage 29, 46–53 10.1016/j.neuroimage.2005.03.046 16085428

[B60] Roettger T. Domahs U. Grande M. Domahs F. (2012). Structural factors affecting the assignment of word stress in German. J. Ger. Linguist. 24, 53–94 10.1017/S1470542711000262

[B61] Sadato N. Campbell G. Ibánez V. Deiber M. Hallett M. (1996). Complexity affects regional cerebral blood flow change during sequential finger movements. J. Neurosci. 16, 2691–2700 8786445 10.1523/JNEUROSCI.16-08-02691.1996 PMC6578781

[B62] Sakai K. Hikosaka O. Miyachi S. Takino R. Sasaki Y. Pütz B. (1998). Transition of brain activation from frontal to parietal areas in visuomotor sequence learning. J. Neurosci. 18, 1827–1840 9465007 10.1523/JNEUROSCI.18-05-01827.1998 PMC6792634

[B63] Sakurai Y. (1990). Hippocampal cells have behavioral correlates during the performance of an auditory working memory task in the rat. Behav. Neurosci. 104, 253–263 10.1037/0735-7044.104.2.253 2346620

[B64] Sakurai Y. (1994). Involvement of auditory cortical and hippocampal neurons in auditory working memory and reference memory in the rat. J. Neurosci. 14, 2606–2623 8182430 10.1523/JNEUROSCI.14-05-02606.1994 PMC6577488

[B65] Scheperjans F. Eickhoff S. B. Hömke L. Mohlberg L. Hermann K. Amunts K. (2008a). Probabilistic maps, morphometry, and variability of cytoarchitectonic areas in the human superior parietal cortex. Cereb. Cortex 18, 2141–2157 10.1093/cercor/bhm241 18245042 PMC3140197

[B66] Scheperjans F. Hermann K. Eickhoff S. B. Amunts K. Schleicher A. Zilles K. (2008b). Observer-Independent Cytoarchitectonic Mapping of the Human Superior Parietal Cortex. Cereb. Cortex. 18, 846–867 10.1093/cercor/bhm116 17644831

[B67] Schmidt-Kassow M. Roncaglia-Denissen M. Kotz S. A. (2011b). Why pitch sensitivity matters: event-related potential evidence of metric and syntactic violation detection among Spanish late learners of German. Front. Psychol. 2, 1–11 10.3389/fpsyg.2011.00131 21734898 PMC3120976

[B68] Schmidt-Kassow M. Rothermich K. Schwartze M. Kotz S. A. (2011a). Did you get the beat? Late proficient French-German learners extract strong–weak patterns in tonal but not in linguistic sequences. Neuroimage 54, 568–576 10.1016/j.neuroimage.2010.07.062 20692349

[B69] Schneider K. (2007). “Acquisition of word stress in German: vowel duration and incompleteness of closure,” in Proceedings of the ICPhS XVI (Saarbrücken), 1565–1568

[B70] Schneider K. Möbius B. (2007). Word stress correlates in spontaneous child-directed speech in German, in Proceedings of the Interspeech (Antwerp), 1394–1397

[B71] Schwartze M. Rothermilch K. Kotz S. A. (2012a). Functional dissociation of pre-SMA and SMA-proper in temporal processing. Neuroimage 60, 290–298 10.1016/j.neuroimage.2011.11.089 22178297

[B72] Schwartze M. Tavano A. Schröger E. Kotz S. A. (2012b). Temporal aspects of prediction in audition: cortical and subcortical neural mechanisms. Int. J. Psychophysiol. 83, 200–207 10.1016/j.ijpsycho.2011.11.003 22108539

[B73] Scott S. K. Blank C. C. Rosen S. Wise R. J. (2000). Identification of a pathway for intelligible speech in the left temporal lobe. Brain 123, 2400–2406 10.1093/brain/123.12.2400 11099443 PMC5630088

[B74] Shibasaki H. Sadato N. Lyshkow H. Yonekura Y. Honda M. Nagamine T. (1993). Both primary motor cortex and supplementary motor area play an important role in complex finger movement. Brain 116, 1387–1398 10.1093/brain/116.6.1387 8293277

[B75] Stanton H. M. (1928). Seashore measures of musical talent. Psychol. Monogr. Gen. Appl. 39, 135–144 10.1037/h0093342

[B76] Tappeiner E. Domahs U. Domahs F. (2007). Wortakzent im Sprachkontext Deutsch-Italienisch. Zeitschrift für Dialektologie und Linguistik, 74, 266–291

[B77] Tewes U. (1991). HAWIE-R. Hamburg-Wechsler Intelligenstest für Erwachsene. Revision. Bern: Huber

[B78] Van der Hulst H. (ed.). (1999). Word Prosodic Systems in the Languages of Europe. Empirical Approaches to Language Typology, Vol. 4. Berlin; New York: Walter de Gruyter

[B79] Vennemann T. (1990). Syllable structure and simplex accent in modern standard German. CLS 26, 399–412

[B80] Vennemann T. (1991). Syllable structure and simplex accent in Modern Standard German, in Papers from the 26th Regional Meeting of the Chicago Linguistic Society. II. The Parasession on the Syllable in Phonetics and Phonology (CLS 26), eds Ziolkowski M. Noske M. Deaton K. (Chicago, IL: Chicago Linguistic Society), 399–412

[B81] Wiese R. (1996). The Phonology of German. Oxford: Clarendon Press

[B82] Wildgruber D. Hertrich I. Riecker A. Erb M. Anders S. Grodd W. (2004). Dinstinct frontal regions subserve evaluation of linguistic and emotional aspects of speech intonation. Cereb. Cortex 14, 1384–1389 10.1093/cercor/bhh099 15217896

[B83] Wood G. Ischebeck A. Koppelstaetter F. Gotwald T. Kaufmann L. (2009). Developmental trajectories of magnitude processing and interference control: an fMRI study. Cereb. Cortex 19, 2755–2765 10.1093/cercor/bhp056 19357393 PMC2853708

[B84] Yonelinas A. P. (2013). The hippocampus supports high-resolution binding in the service of perception, working memory and long-term memory. Behav. Brain Res. 254, 34–44 10.1016/j.bbr.2013.05.030 23721964 PMC3773061

[B85] Zarate J. M. Zatorre R. J. (2005). Neural substrates governing audiovocal integration for vocal pitch regulation in singing. Ann. N.Y. Acad. Sci. 1060, 404–408 10.1196/annals.1360.058 16597793

[B86] Zilles K. Rehkämper G. (1998). Funktionelle Neuroanatomie (3. komplett überarbeitete und aktualisierte Auflage). Berlin: Springer-Verlag

