# Supplementary material for: Processing word prosody—behavioral and neuroimaging evidence for heterogeneous performance in a language with variable stress
Source: Front Psychol. 2014 Apr 29;5:365. doi: 10.3389/fpsyg.2014.00365 (PMC4010785; doi:10.3389/fpsyg.2014.00365)
Supplement: Supplementary file 1 [file DataSheet1.PDF]

Supplemental material:

Accuracy per group and condition.

Participant	Sex	Group	Accuracy segmental [%]	Accuracy suprasegmental [%]
1	m	above median	84,4	100,0
2	m	above median	56,3	96,9
3	f	above median	71,9	93,8
4	f	above median	96,9	93,8
5	f	above median	93,8	90,6
6	m	above median	93,8	87,5
7	m	above median	71,9	87,5
8	m	above median	81,3	84,4
9	m	above median	93,8	84,4
10	f	above median	84,4	84,4
11	f	above median	71,9	81,3
12	m	above median	75,0	78,1
13	m	above median	68,8	78,1
14	f	below median	87,5	68,8
15	m	below median	78,1	68,8
16	m	below median	40,6	68,8
17	m	below median	84,4	65,6
18	f	below median	84,4	65,6
19	f	below median	96,9	65,6
20	m	below median	71,9	62,5
21	m	below median	71,9	62,5
22	m	below median	84,4	62,5
23	m	below median	87,5	59,4
24	m	below median	65,6	59,4
25	f	below median	84,4	56,3
